# Exploring the Effects of Genetic Variants on Clinical Profiles of Parkinson’s Disease Assessed by the Unified Parkinson’s Disease Rating Scale and the Hoehn–Yahr Stage

**DOI:** 10.1371/journal.pone.0155758

**Published:** 2016-06-14

**Authors:** Chen Shi, Zheng Zheng, Qi Wang, Chaodong Wang, Dabao Zhang, Min Zhang, Piu Chan, Xiaomin Wang

**Affiliations:** 1 Department of Neurobiology, School of Basic Medical Sciences, Capital Medical University, Beijing, China; 2 Bioinformatics Center, School of Biomedical Engineering, Capital Medical University, Beijing, China; 3 Department of Neurobiology, Beijing Institute of Geriatrics, Xuanwu Hospital of Capital Medical University, Beijing, China; 4 Department of Statistics, Purdue University, West Lafayette, Indiana, United States of America; 5 Department of Neurology, Beijing Institute of Geriatrics, Xuanwu Hospital of Capital Medical University, Beijing, China; 6 Key Laboratory on Neurodegenerative Disease of Ministry of Education and Key Laboratory on Parkinson’s Disease of Beijing, Beijing, China; 7 Department of Neurology, The Affiliated Sanming First Hospital of Fujian Medical University, Sanming, Fujian, China; 8 Parkinson Disease Center of Beijing Institute for Brain Disorders, Capital Medical University, Beijing, China; 9 Alzheimer Disease Center of Beijing Institute for Brain Disorders, Capital Medical University, Beijing, China; Duke University, UNITED STATES

## Abstract

Many genetic variants have been linked to familial or sporadic Parkinson’s disease (PD), among which those identified in *PARK16*, *BST1*, *SNCA*, *LRRK2*, *GBA* and *MAPT* genes have been demonstrated to be the most common risk factors worldwide. Moreover, complex gene-gene and gene-environment interactions have been highlighted in PD pathogenesis. Compared to studies focusing on the predisposing effects of genes, there is a relative lack of research investigating how these genes and their interactions influence the clinical profiles of PD. In a cohort consisting of 2,011 Chinese Han PD patients, we selected 9 representative variants from the 6 above-mentioned common PD genes to analyze their main and epistatic effects on the Unified Parkinson’s Disease Rating Scale (UPDRS) and the Hoehn and Yahr (H-Y) stage of PD. With multiple linear regression models adjusting for medication status, disease duration, gender and age at onset, none of the variants displayed significant main effects on UPDRS or the H-Y scores. However, for gene-gene interaction analyses, 7 out of 37 pairs of variants showed significant or marginally significant associations with these scores. Among these, the *GBA* rs421016 (L444P)×*LRRK2* rs33949390 (R1628P) interaction was consistently significant in relation to UPDRS III and UPDRS total (I+II+III), even after controlling for the family-wise error rate using False Discovery Rate (FDR-corrected p values are 0.0481 and 0.0070, respectively). Although the effects of the remaining pairs of variants did not survive the FDR correction, they showed marginally significant associations with either UPDRS or the H-Y stage (raw p<0.05). Our results highlight the importance of epistatic effects of multiple genes on the determination of PD clinical profiles and may have implications for molecular classification and personalized intervention of the disease.

## Introduction

Parkinson’s disease (PD) is the second most prevalent neurodegenerative disorder after Alzheimer’s disease. It affects more than 1% of individuals over the age of 60 and has caused increasingly prevalent social and economic burdens among a rapidly aging world population. PD is a progressive neurological disorder characterized by a series of motor and non-motor symptoms such as resting tremor, rigidity, bradykinesia, postural instability, and autonomic dysfunction[1]. The pathological hallmark of PD involves the selective loss of dopaminergic neurons in the substantia nigra. Cell loss in other areas of the central and peripheral nervous system are also well documented[2]. Although PD was first described by James Parkinson in 1817, the etiology of PD remains elusive and there is neither a definitive diagnostic test nor a validated biomarker available[3] to test for disease onset and progression.

Recent studies have highlighted the importance of genetic factors in the etiology of PD. Thus far, over 30 genes/loci have been linked to familial or sporadic PD. Among these genes/loci, mutations or polymorphisms in the *PARK16*, *BST1*, *SNCA*, *LRRK2*, *GBA* and *MAPT* genes have been demonstrated to be the most common risk factors for the disorder worldwide and have been repeatedly validated in diverse ethnicities[4–12]. Moreover, due to the shared mechanisms and concurrence of multiple genes related to PD in brain pathology[13–15], complex gene-gene and gene-environment interactions have been noted in PD pathogenesis[16–21].

While most studies focus on the genetic variants as risk factors for PD, there is a lack of research investigating the relationship of these variants and their interactions with the clinical profiles of PD, such as age at onset and quantitative clinical evaluations with the Unified Parkinson’s Disease Rating Scale (UPDRS) and the Hoehn and Yahr (H-Y) stage. In the current study, we have analyzed 9 representative polymorphisms that reside in the 6 common PD related genes mentioned above. Using the data collected from the Chinese National Consortium on Neurodegenerative Diseases (CNCND), we tested their independent associations with UPDRS and the H-Y stage, and also the effects of pairwise interactions between these polymorphisms on both UPDRS and the H-Y stage of PD.

## Materials and Methods

### Ethics statement

Research conducted in this study was approved by the Ethics Committee of Xuanwu Hospital of Capital Medical University and samples were collected with the understanding and written consent of the subjects or their legal guardians.

### Study cohort

The study subjects were derived from our previous genetic study cohort[22]. All PD cases in this study were sporadic, and collected and registered in the PD cohort of the Chinese National Consortium on Neurodegenerative Disease (CNCPD, www.chinapd.cn). The consortium was based on a large-scale collaboration between 42 clinical centers and managed by the coordination center at Xuanwu Hospital of Capital Medical University in Beijing. PD was diagnosed by at least two movement disorders specialists using the United Kingdom PD Society Brain Bank Criteria[23]. Patients with a family history of PD in a first- or second- degree relative were not included. Patients meeting these criteria were enrolled between January 2008 and April 2010, during which time tissue samples were collected for DNA analysis.

### Demographic data and clinical assessments

Demographic data (gender, age at onset, medication status, disease duration, etc.) were collected and clinical rating measures (UPDRS total, I, II, III and the H-Y stage) of PD symptoms were performed by at least two PD specialists in each center and monitored by the coordination center at Xuanwu Hospital. The scale for each question in UPDRS is rated from 0 to 4 (0 = normal; 1 = slight; 2 = mild; 3 = moderate; 4 = severe), while the value for the H-Y stage ranges from 1 to 5 (a higher score indicates more severe impairment and disability). Additional detailed information of the data was reported in earlier studies[22, 24]. All the clinical ratings were examined in the OFF state following the same protocol.

### Selection and genotyping of genetic variants

For genotyping of PD patients, we selected 9 representative variants from 6 globally common PD-associated genes with repeatedly validated evidence in both Chinese and other ethnicities. The rationale and strategy for selection was elaborated in our previous genetic study[22]. Briefly, rs421016 (L444P) is the most common *GBA* variant identified in Asian PD[25]; rs823144 is the tag SNP for the linkage disequilibrium (LD) block containing the most significant PD-associated SNPs (rs947211, rs823128, rs823156, rs708730) identified in the *PARK16* locus in both Asian and European genome-wide association (GWA) studies[5, 6]; rs4273468 is a tag SNP for the most significant PD-associated SNPs (rs11931532 and rs4538475) identified on the *BST1* locus in several recent GWA studies[5]; rs356219 is among the most common SNPs identified in *SNCA* associated with PD in Chinese Han population[26]; the *SNCA* Rep1 allele is a widely evidenced predisposing factor for PD worldwide[27]; rs33949390 (R1628P) and rs34778348 (G2385R) were the sole Asian-type variants of *LRRK2*, the most popular PD gene[28, 29]; rs242562 and rs2435207 in *MAPT* were selected because they were the only two H1-specific *MAPT* polymorphisms associated with PD[30, 31], and the association of the complete H1 haplotype with PD was absent in Asians[32]. The experimental procedure of genotyping was presented in previous study[22]. Oligo 6.0 (Molecular Biology Insights, Inc. CO, USA) and primer 5.0 software (Premier, Canada) were employed to design the polymerase-chain-reaction (PCR) assays and extension primers for variants. ABI 3730 DNA analyzer (Applied Biosciences, Inc, CT, USA) was utilized to sequence the PCR products. The Chromas 1.45 software was used for sequence reading. The genotype of each single nucleotide polymorphism (SNP) is based on the minor allele count except for the repeated sequence of *SNCA* Rep1. *SNCA* Rep1 was coded separately as 259, 261 and 263 on the basis of the length of PCR products[33]. Detailed information for each variant is presented in Table 1. Importantly, for LRRK2 and GBA, previous studies have demonstrated that even if the entire genes had been examined, few PD-associated mutations/variants would be identified[34, 35].

**Table 1:** Information for genetic variants selected for genotyping.

Variant	Chr	Location ^a^	Gene	Minor/Major Allele
rs421016	1	155235252	GBA	C/T
rs823144	1	205775418	PARK16	C/A
rs4273468	4	15736240	BST1	G/A
rs356219	4	89716450	SNCA	A/G
Rep1 allele	4	3’-UTR	SNCA	263/261/259 bp
rs33949390	12	40320043	LRRK2	G/C
rs34778348	12	40363526	LRRK2	A/G
rs242562	17	45949373	MAPT	G/A
rs2435207	17	45981562	MAPT	A/G

Chr: chromosome.

^a^Based on GRCh38, the Genome Reference Consortium Human genome build 38 published on May 7, 2014.

### Statistical analysis

UPDRS I acts as a general screening tool for the presence of non-motor symptoms of PD and cannot be used to adequately measure the severity of such syndromes[36]. Additionally, UPDRS II is the subjective patient-derived assessment of motor symptoms while UPDRS III is the physician-derived assessment of motor symptoms[37]. The H-Y stage, originally designed as a descriptive staging score, is another popular estimate of the functional deficits and objective signs of PD. As such, UPDRS III and UPDRS total (I+II+III) as well as the H-Y stage were used as independent clinical variables in our analysis. For linear and non-parametric analyses, we presented the values of UPDRS and H-Y stage as both means and standard deviation (SD), as well as median and interquartile range (IQR), respectively.

A Fisher’s exact test was employed for evaluating the Hardy-Weinberg Equilibrium (HWE) of each genetic variant. For multi-factorial analysis, homozygotes and heterozygotes for the major and minor alleles of each variant were introduced under the co-dominant model[38]. We then standardized all the genetic terms and the four covariates, including disease duration (continuous variable), onset age (continuous variable), gender (binary variable), and medication status (binary variable), to bring all variables into proportion with one another. Multiple linear regression models were constructed for analyzing the main effect of each genetic variant on UPDRS III, UPDRS total (I+II+III) and the H-Y stage by comparing model 1 (full model including the main effect of each genetic variant and the 4 covariates) and model 2 (reduced model with only 4 covariates). In terms of gene-gene interactions, all possible pairs of genetic variants from different genes were considered. For each pair we fitted two multiple regression models where one included only the main effects of both variants and the other model included both the main and epistatic effects between the two variants. Both models were adjusted for the above-mentioned clinical covariates. A general linear test with four degrees of freedom was employed to test for the significance of the interaction effects on the basis of these two models.

Two-sided tests were considered for all hypotheses. In addition to the uncorrected p values, the false discovery rate (FDR) was used to control the family-wise type I error rate and an FDR adjusted p value less than 0.05 was determined to be statistically significant[39]. Data preprocessing and analyses were performed using PLINK[40] and the statistical package SAS (version 9.3; SAS Institute Inc., Cary, NC).

## Results

All the subjects in this study were ethnic Chinese Hans. Table 2 summarizes the demographic information of all subjects in the study. Among the 2,011 PD patients, scores for UPDRS and the H-Y stage were available in 1,622 and 1,587 PD patients, respectively.

**Table 2:** Demographic characteristics of study participants.

Characteristics	PD patients (n = 2,011)
**Gender, n (%)**	
Male	1,231 (61.2)
Female	780 (38.8)
**Age at collection, mean ± SD** ^a^	62.7 ± 10.1
**Age at onset, mean ± SD** ^a^	58.6 ± 10.4
≤50 years, n (%)	1318 (79.0)
>50 years, n (%)	351 (21.0)
**Disease duration, n (%)**	
≤2 years	540 (32.1)
2–5 years	684 (40.7)
5–10 years	330 (19.6)
>10 years	127 (7.6)
**Medication, n (%)**	
Yes	913 (45.4)
No	1,098 (54.6)
**UPDRS total (n = 1,622)**	
Mean ± SD ^a^	36.13 ± 20.6
Median (IQR) ^b^	32 (24)
**UPDRS I (non-motor experiences of daily living)**	
Mean ± SD ^a^	2.11 ± 2.09
Median (IQR) ^b^	2 (3)
**UPDRS II (self-evaluation of the activities of daily life)**	
Mean ± SD ^a^	10.56 ± 6.30
Median (IQR) ^b^	9 (6)
**UPDRS III (evaluation of motor examination)**	
Mean ± SD ^a^	23.46 ± 14.15
Median (IQR) ^b^	21 (18)
**Hoehn-Yahr (n = 1,587)**	
Mean ± SD ^a^	1.95 ± 0.76
Median (IQR) ^b^	2 (1.5)

UPDRS: Unified Parkinson’s disease rating scale; Hoehn-Yahr: the Hoehn and Yahr stage.

Continuous variables were presented as mean ± SD; discrete variables were presented as numbers (%).

^a^SD: standard deviation.

^b^IQR: interquartile range.

For UPDRS and the H-Y stage, both mean with SD and median with IQR were reported.

### Main effects

The HWE test excluded *BST1* rs4273468 (p<0.0001), leaving the other 8 genetic variants for subsequent analyses. Since *SNCA* Rep 1 has 3 alleles (259/261/263 bp), it was coded and analyzed separately. Therefore, the total number of variants analyzed was 10. Using the multiple linear regression models, we tested the main effects of each variant on UPDRS III, UPDRS total (I+II+III) and the H-Y stage. The results suggested that none of the main effects of these variants reached statistical significance, even before the FDR-correction (see S1–S3 Tables). Considering the uneven distribution of UPDRS and the discrete values of the H-Y stage, we also performed non-parametric analysis using Kruskal-Wallis rank sum test for the association between each genetic variant with the UPDRS scores and the H-Y stage. As a result, all the p values for such tests were >0.05 (S4 Table), consistent with the linear regression analyses.

### Epistatic effects

We next tested the interactive effects between variants from different genes on the clinical symptoms, in a multivariate linear model adjusting disease duration, age at onset, gender, and medication status. A total of 37 pairs of variants were evaluated for their associations with UPDRS III, UPDRS total (I+II+III) and the H-Y stage, respectively. As a result, 7 pairs of interactive variants showed statistical significance or marginal significance in association with UPDRS or the H-Y stage of PD, as shown in Table 3.

**Table 3:** Significant and marginally significant interaction effects on UPDRS III, UPDRS total (I+II+III) and the H-Y stage.

Clinical scores	Variant-pairs	Raw p value^a^	FDR adjusted p value
**UPDRS III**	**GBA rs421016 × LRRK2 rs33949390**	**0.0013**	**0.0481**
	PARK16 rs823144 **×**LRRK2 rs33949390	0.0411	0.7605
**UPDRS total (I+II+III)**	**GBA rs421016 × LRRK2 rs33949390**	**0.0002**	**0.0070**
	PARK16 rs823144 × LRRK2 rs33949390	0.0445	0.6253
**Hoehn-Yahr**	GBA rs421016 × LRRK2 rs33949390	0.0056	0.2088
	SNCA rs356219 × MAPT rs242562	0.0466	0.5749
	SNCA Rep259 × LRRK2 rs33949390	0.0394	0.5749

^a^Raw p values compared interaction effects between model1 (containing only main effects of two variants) and model 2 (including both main effects and interactions of two variants). All the regressions adjusted for medication status, disease duration, age at onset, and gender.

The bolded numbers indicate significance after controlling for the family-wise error rate using FDR at 0.05.

The *GBA* rs421016 (L444P)×*LRRK2* rs33949390 (R1628P) interaction was consistently significant in relation to UPDRS III and UPDRS total (I+II+III), even after controlling for the family-wise error rate (FDR-adjusted p values were 0.0481 and 0.0070, respectively), and also displayed a marginally significant association with the H-Y stage (raw p = 0.0056).

Although the effects of no other pairs of variants survived the FDR correction, several interactions showed marginal association with UPDRS or the H-Y stage. Among these interactions, *PARK16* rs823144×*LRRK2* rs33949390 was marginally associated with both UPDRS III and UPDRS total (raw p value was 0.0411 and 0.0445, respectively). In addition, *SNCA* rs356219×*MAPT* rs242562 and *LRRK2* rs33949390×*SNCA* Rep259 were found to marginally interact with the H-Y stage (raw p values were 0.0466 and 0.0394, respectively). Moreover, it is interesting to note that *LRRK2* R1628P was involved in 6 out of 7 significant or marginally significant pairs of epistatic effects with other PD related genes.

## Discussion

As the worldwide aging population continues to increase, neurodegenerative diseases such as PD have been a popular field of study for both clinical and basic research. As an incurable disease, PD is progressive by definition but the course of the progression is not linear[41, 42]. Therefore, it becomes crucial to understand the basis of clinical symptoms and predict the course of disease progression. To explore the genetic basis underlying the clinical symptoms of PD, we evaluated the association between the selected PD susceptibility variants and UPDRS as well as the H-Y scale, using the largest PD dataset of Han Chinese to date. In contrast to reports by others, none of the 9 variants had significant effect on UPDRS or the H-Y stage in our study. For instance, Alcalay et al. found no difference in clinical phenotype including UPDRS between *LRRK2* G2019S mutation carriers and non-carriers[43], while Pulkes et al. observed poorer H-Y stage in *LRRK2* R1628P carriers than non-carriers in the Thai population[44]. Similar inconsistences were also noted in the Chinese population. While one study demonstrated no difference in UPDRS and the H-Y stage between *GBA* L444P carriers and non-carriers[25], another reported higher UPDRS in *GBA*-related PD patients than in *LRRK2*-related and sporadic patients carrying none of those variants[24]. Although the explanations for such conflicting results remain largely unclear, it reflects considerable variation in the rate of neurodegeneration among individuals[45], and confirms the existence of multiple determinants for PD clinical profiles[46, 47], in which the effect of a single gene or locus may be modest.

Although there have been expanding data supporting the important roles of gene-gene and gene-environment interactions in determining PD susceptibility[16–21], it is yet unclear how these interactions influence the clinical features (e.g. severity and progression) of PD. In this study, we identified 7 pairs of significant or marginally significant gene-gene interactions in relation to UPDRS III and UPDRS total (I+II+III), as well as the H-Y stage. Among these, the interactive effects of *LRRK2* R1628P×*GBA* L444P on UPDRS and the H-Y stage were the most significant, maintaining statistical significance even after FDR correction. The remaining pairs, though not surviving the FDR correction, displayed marginal effects on UPDRS or the H-Y stage. These results suggest that multiple genetic factors with minimal individual effects can display strong synergistic effects on PD clinical profiles. In addition, the association of these pairs with the H-Y stage seems weaker than that with UPDRS. The explanations for such a difference may be multifold. First, the H-Y stage is a simple descriptive staging scale designed for single-item assessments of merely motor function, while UPDRS is a much more comprehensive scale covering both motor and non-motor aspects as well as daily activities and complications, and is therefore more sensitive by nature than the H-Y stage. Second, the H-Y stage is a brief assessment performed by the examiner and is dependent on personal judgment. It is more subjective as it can be influenced by the variation in training and clinical experience of site neurologists.

Variants in *LRRK2* are recognized as the most common genetic cause of both familiar and sporadic PD[48]. The two missense variants (R1628P and G2385R) of *LRRK2* and the missense mutation (L444P) of *GBA* have been established to be the most important genetic risk factors for PD in Asians[25, 49, 50]. Since both *LRRK2* and *GBA* participate in the lysosomal-autophagy pathway involved in PD pathogenesis[51] and mutation carriers for both genes display the presence of Lewy bodies in the PD brain[52, 53], our results suggest that the *LRRK2-GBA* interaction may facilitate the autophagy-dependent impairment and the accumulation of α-Synuclein in dopaminergic neurons. Moreover, it is interesting to note that most (6/7) of the interactive pairs displaying significant or marginal associations with UPDRS or the H-Y stage included *LRRK2* R1628P, indicating that *LRRK2* may serve as a central genetic regulator of PD clinical profiles. As *LRRK2* is an unusually large protein with 2,527 amino acids and harbors several interaction domains, it may work as a scaffold for multiple protein partners[54]. However, further studies are needed to explore such interactions at protein levels to affirm their roles in the PD pathology and manifestations.

In this study, restricting the interaction analyses to 9 representative variants allows us to reduce the burden of multiple testing and may provide a simplified model for delineating an interactive picture of PD genes[55]. However, given the more complicated crosstalk between genes and the generally larger sample size requirement of gene-gene interaction studies, the results must be interpreted with caution. Future studies require the inclusion of more genetic variants and the integration of other information, such as gene expression profiles, epigenetic data and environmental exposures, to replicate our results and disclose the determinants of PD clinical profiles.

In conclusion, our study suggests that individual genetic variants may not have enough influence on clinical profiles of PD, while multiple genetic factors can work synergistically to determine disease severity and progression. These results provide further evidence supporting the multifactorial etiology of PD and may have implications in molecular classification and personalized intervention of the disease.

## Supporting Information

S1 Table Testing for marginal effect of each genetic variant on UPDRS III. (XLSX)

S2 Table Testing for marginal effect of each genetic variant on UPDRS total (I+II+III). (XLSX)

S3 Table Testing for marginal effect of each genetic variant on H-Y. (XLSX)

S4 Table Testing for non-parametric analysis of genetic-clinical correlation. (XLSX)
